# Supplementary material for: Protocol for a randomized crossover study of thigh cuff inflation in experimental hemorrhage: Assessing its potential as a model for zone 3 REBOA
Source: PLoS One. 2025 Jul 28;20(7):e0327404. doi: 10.1371/journal.pone.0327404 (PMC12303282; doi:10.1371/journal.pone.0327404)

PLOS ONE

Hemodynamic effects of thigh cuff inflation in experimental hemorrhage: Protocol for a randomized, controlled crossover study

--Manuscript Draft--

**Manuscript Number:**	PONE-D-24-58304
**Article Type:**	Study Protocol
**Full Title:**	Hemodynamic effects of thigh cuff inflation in experimental hemorrhage: Protocol for a randomized, controlled crossover study
**Short Title:**	Hemodynamic effects of thigh cuff inflation in experimental hemorrhage
**Corresponding Author:**	Sara Stadskleiv Torbjørnsen<br>University of Oslo Faculty of Medicine: Universitetet i Oslo Det medisinske fakultet<br>Oslo, NORWAY
**Keywords:**	Cardiovascular physiology; Hemodynamics; Trauma; Noncompressible hemorrhage; Hemorrhagic resuscitation; REBOA; Traumatic brain injuriy; Cerebral perfusion; Experimental model; Study Protocol; Lower body negative pressure; Thigh cuff inflation; RCT
**Abstract:**	Resuscitative endovascular balloon occlusion of the aorta (REBOA) aims to control noncompressible hemorrhage in trauma patients. REBOA has mainly been studied in animals and trauma patients. We aim to study whether thigh cuff inflation combined with simulated hemorrhage can serve as an experimental human model of zone 3 REBOA.<br>Twenty healthy volunteers will be exposed to bilateral proximal thigh cuff inflation to suprasystolic pressures to mimic the hemodynamic effects of REBOA during simulated hemorrhage using lower body negative pressure. Each volunteer will undergo two experimental conditions in a randomized order during the same visit. In one condition, volunteers will be exposed to 60 mmHg LBNP for six minutes. In the other condition, 60 mmHg LBNP will be applied for six minutes, with addition of thigh cuff inflation during the last three minutes. Systemic hemodynamic parameters will be measured continuously and non-invasively, including arterial blood pressure, stroke volume, and heart rate. Middle cerebral artery blood velocity and cerebral oxygenation will be measured as indicators of cerebral hemodynamics. The effect of thigh cuff inflation on systemic and cerebral hemodynamics will be assessed using mixed-effects regression analyses. Pain associated with thigh cuff inflation will be assessed using a verbal numerical rating scale.<br>This study aims to examine the systemic and cerebral hemodynamic effects of combined thigh cuff inflation and lower body negative pressure in healthy volunteers. Based on the feasibility and findings, the potential of this combination as a model for zone 3 REBOA in simulated hemorrhage will be discussed.
**Order of Authors:**	Sara Stadskleiv Torbjørnsen, MD-student
	Sole Lindvåg Lie, MD, PhD
	Marius Rehn, MD, PhD
	Jonny Hisdal, MSC, PhD
	Lars Øivind Høiseth, MD, PhD
**Additional Information:**	
**Question**	**Response**
**Financial Disclosure**<br><br>Enter a financial disclosure statement that describes the sources of funding for the work included in this submission. Review the submission guidelines for detailed	The author(s) received no specific funding for this work.

Powered by Editorial Manager® and ProduXion Manager® from Aries Systems Corporation

requirements. View published research articles from *PLOS ONE* for specific examples.<br><br>This statement is required for submission and **will appear in the published article** if the submission is accepted. Please make sure it is accurate.<br><br>**Funded studies**<br>Enter a statement with the following details:<br>• Initials of the authors who received each award<br>• Grant numbers awarded to each author<br>• The full name of each funder<br>• URL of each funder website<br>• Did the sponsors or funders play any role in the study design, data collection and analysis, decision to publish, or preparation of the manuscript?<br><br>Did you receive funding for this work?	
**Competing Interests**<br><br>Use the instructions below to enter a competing interest statement for this submission. On behalf of all authors, disclose any competing interests that could be perceived to bias this work—acknowledging all financial support and any other relevant financial or non-financial competing interests.<br><br>This statement is required for submission and **will appear in the published article** if the submission is accepted. Please make sure it is accurate and that any funding sources listed in your Funding Information later in the submission form are also declared in your Financial Disclosure statement.<br><br>View published research articles from *PLOS ONE* for specific examples.	The authors have no competing interests.

**NO authors have competing interests**<br><br>Enter: *The authors have declared that no competing interests exist.*<br><br>**Authors with competing interests**<br><br>Enter competing interest details beginning with this statement:<br><br>*I have read the journal's policy and the authors of this manuscript have the following competing interests: [insert competing interests here]*<br><br>* typeset	
**Ethics Statement**<br><br>Enter an ethics statement for this submission. This statement is required if the study involved:<br><br>• Human participants<br>• Human specimens or tissue<br>• Vertebrate animals or cephalopods<br>• Vertebrate embryos or tissues<br>• Field research<br><br>Write "N/A" if the submission does not require an ethics statement.<br><br>General guidance is provided below. Consult the submission guidelines for detailed instructions. **Make sure that all information entered here is included in the Methods section of the manuscript.**	Human Subject Research:<br>The Regional Ethics Committee (REK South East D, ref 522416) and the the Data Protection Officer at Oslo University Hospital (ref.nr: 22/22307) have assessed and approved this protocol. The institution responsible for this evaluation was the University Hospital of Oslo, Norway.<br>All volunteers in this study must give written and oral consent before participating.

Powered by Editorial Manager® and ProduXion Manager® from Aries Systems Corporation

**Format for specific study types**<br><br>**Human Subject Research (involving human participants and/or tissue)**<br>• Give the name of the institutional review board or ethics committee that approved the study<br>• Include the approval number and/or a statement indicating approval of this research<br>• Indicate the form of consent obtained (written/oral) or the reason that consent was not obtained (e.g. the data were analyzed anonymously)<br><br>**Animal Research (involving vertebrate animals, embryos or tissues)**<br>• Provide the name of the Institutional Animal Care and Use Committee (IACUC) or other relevant ethics board that reviewed the study protocol, and indicate whether they approved this research or granted a formal waiver of ethical approval<br>• Include an approval number if one was obtained<br>• If the study involved *non-human primates*, add *additional details* about animal welfare and steps taken to ameliorate suffering<br>• If anesthesia, euthanasia, or any kind of animal sacrifice is part of the study, include briefly which substances and/or methods were applied<br><br>**Field Research**<br><br>Include the following details if this study involves the collection of plant, animal, or other materials from a natural setting:<br>• Field permit number<br>• Name of the institution or relevant body that granted permission	
**Data Availability**<br><br>Authors are required to make the data underlying their research findings fully available, without restriction.<br><br>If the manuscript reports pilot or preliminary results, the data underlying those results must be made available at the time of publication. A Data Availability	N/A - No results are reported

Powered by Editorial Manager® and ProduXion Manager® from Aries Systems Corporation

Statement describing where the data can be found is required at submission. Your answers to this question constitute the Data Availability Statement and will be published in the article, if accepted. When legal or ethical requirements prohibit public data sharing, authors must indicate how researchers may obtain access to the data. See the PLOS Data Policy and FAQ for detailed information.<br><br>**Important:** Stating 'data available on request from the author' is not sufficient. If your data are only available upon request, select 'No' for the first question and explain your situation in the text box.<br><br>For protocols without pilot or preliminary data, authors are strongly encouraged to state how they plan to share research data from their study when it is completed or published.<br><br>If your study includes pilot data, will they be made fully available without restriction?	
**Describe where the data may be found in full sentences. If you are copying our sample text, replace any instances of XXX with the appropriate details.**<br><br>• If **no pilot data are reported** in the manuscript, please enter the following: *No datasets were generated or analysed during the current study. All relevant data from this study will be made available upon study completion.*<br>• If the manuscript reports pilot data **held in a public repository**, include URLs, accession numbers or DOIs. If this information will only be available after acceptance, indicate this by ticking the box below. For example: *All XXX files are available from the XXX database (accession number(s) XXX, XXX.).*<br>• If the manuscript reports pilot data contained **within the manuscript and/or**	No datasets were generated or analysed during the current study. All relevant data from this study will be made available upon study completion.

**Supporting Information files**, enter the following:<br>*All relevant data are within the manuscript and its Supporting Information files.*<br>• If neither of these applies but you are able to provide **details of access elsewhere**, with or without limitations, please do so. For example:<br><br>*Data cannot be shared publicly because of [XXX]. Data are available from the XXX Institutional Data Access / Ethics Committee (contact via XXX) for researchers who meet the criteria for access to confidential data.*<br><br>*The data underlying the results presented in the study are available from (include the name of the third party and contact information or URL).*<br>• This text is appropriate if the data are owned by a third party and authors do not have permission to share the data.<br><br>For study protocols with data management or sharing plans, authors are encouraged to briefly describe in their Data Availability Statement how data they generate will be made accessible when the study is completed. For example:<br>• Deidentified research data will be made publicly available when the study is completed and published.<br><br>* typeset	
Additional data availability information:	

Powered by Editorial Manager® and ProduXion Manager® from Aries Systems Corporation

Study Protocol Manuscript

Click here to access/download;Manuscript;STUDY_PROTOCOL_2.02.docx

Hemodynamic effects of thigh cuff inflation in experimental hemorrhage: Protocol for a randomized, controlled crossover study

Sara Stadskleiv Torbjørnsen[1, 2], Sole Lindvåg Lie[2, 4], Marius Rehn[1, 3, 4], Jonny Hisdal[1, 2], Lars Øivind Høiseth[1, 5]

[1] Faculty of Medicine, Institute of Clinical Medicine, University of Oslo, Oslo, Norway.

[2] Section of Vascular Investigations, Heart-, lung- and vascular clinic, Oslo University Hospital, Oslo, Norway.

[3] Air Ambulance Department, Division of Prehospital Services, Oslo University Hospital, Oslo, Norway

[4] Department of Research and Development, Norwegian Air Ambulance Foundation, Oslo, Norway,

[5] Department of Anesthesia and Intensive Care Medicine, Division of Emergencies and Critical Care, Oslo University Hospital, Oslo, Norway.

Metadata

Funding

This study is funded by the University of Oslo, the Norwegian Air Ambulance Foundation and Oslo University Hospital.

Competing interests

The authors have no competing interests.

Data availability

The dataset will be available upon publication of the original article.

Abstract

Resuscitative endovascular balloon occlusion of the aorta (REBOA) aims to control noncompressible hemorrhage in trauma patients. REBOA has mainly been studied in animals and trauma patients. We aim to study whether thigh cuff inflation combined with simulated hemorrhage can serve as an experimental human model of zone 3 REBOA.

Twenty healthy volunteers will be exposed to bilateral proximal thigh cuff inflation to suprasystolic pressures to mimic the hemodynamic effects of REBOA during simulated hemorrhage using lower body negative pressure. Each volunteer will undergo two experimental conditions in a randomized order during the same visit. In one condition, volunteers will be exposed to 60 mmHg LBNP for six minutes. In the other condition, 60 mmHg LBNP will be applied for six minutes, with addition of thigh cuff inflation during the last three minutes. Systemic hemodynamic parameters will be measured continuously and non-invasively, including arterial blood pressure, stroke volume, and heart rate. Middle cerebral artery blood velocity and cerebral oxygenation will be measured as indicators of cerebral hemodynamics. The effect of thigh cuff inflation on systemic and cerebral hemodynamics will be assessed using mixed-effects regression analyses. Pain associated with thigh cuff inflation will be assessed using a verbal numerical rating scale.

This study aims to examine the systemic and cerebral hemodynamic effects of combined thigh cuff inflation and lower body negative pressure in healthy volunteers. Based on the feasibility and findings, the potential of this combination as a model for zone 3 REBOA in simulated hemorrhage will be discussed.

Introduction

Among patients under the age of 45 years, trauma is the leading cause of death [1], with the majority of deaths occurring in the prehospital phase [2]. In both civilian and military settings, noncompressible torso hemorrhage (NCTH) represents the majority of potentially preventable trauma deaths [3,4]. Further treatment options are needed to improve the pre- and in-hospital survival of patients with NCTH.

Resuscitative endovascular balloon occlusion of the aorta (REBOA) is a method used in NCTH and hemorrhagic shock as a temporary measure before definitive hemorrhagic control can be achieved [5]. In REBOA, a balloon is inflated in the descending aorta, thereby occluding blood flow and preventing further hemorrhage distal to the occlusion. The balloon can be positioned in three different zones in the descending aorta, depending on the location of the bleeding. Occlusion distal to the renal arteries is designated as zone 3 [6]. Since the introduction of REBOA in the 1950s, REBOA has more recently also been applied in the prehospital setting [7,8].

REBOA has profound hemodynamic effects [9]. Cardiac afterload and arterial blood pressure increase, which may increase cerebral perfusion pressure [10,11]. In addition to systemic hemodynamic effects, cerebral hemodynamic effects are also relevant to investigate since polytrauma patients may suffer from traumatic brain injury (TBI), thus having an increased risk of in intracranial hemorrhage, cerebral edema and increased intracranial hemorrhage, which might worsen the outcome [12,13]. Until now, REBOA has mainly been studied in anesthetized animals, pre-hospital cardiac arrest, hemorrhagic shock and during surgery [14]. However, there is a need for an experimental model to investigate the hemodynamic effects of REBOA in awake humans.

Lower body negative pressure (LBNP) is a model to simulate hemorrhage in awake healthy volunteers. Negative tressure is applied to the lower body, causing pooling of venous blood in the lower extremities and pelvis. Consequently, venous return and cardiac stroke volume are reduced, similar to what is seen during hemorrhage [15]. Thigh cuff inflation is a method to occlude arterial blood flow to the lower extremities [16] and may therefore mimic some of the hemodynamic effects of a zone 3 REBOA. Arterial occlusion of the lower limbs has been shown to temporarily increase MAP, HR and systemic vascular resistance (SVR) [17].

Whether combining thigh cuff inflation during LBNP is a feasible method to investigate the hemodynamic effects of zone 3 REBOA during hemorrhage in healthy volunteers remains to be studied. The aim of this study is to investigate the systemic and cerebral hemodynamic effects of thigh cuff inflation during LBNP in healthy volunteers. The primary hypothesis is that thigh cuff inflation will affect cardiac stroke volume during LBNP. The secondary hypothesis is that thigh cuff inflation will affect the response in mean arterial blood pressure (MAP) and middle cerebral artery velocity (MCAV) during LBNP.

Materials and methods

Study design

This is an ongoing single-center, experimental, randomized, controlled crossover study**Fig 1**. Subject recruitment began on April 4, 2024. Recruitment and data collection is expected to be completed by February 28, 2025. The results are expected to be available by December 1, 2025. Each subject will complete two experimental conditions in a randomized order during a single visit. During one condition, 60 mmHg of LBNP will be applied for six minutes. During the other condition, 60 mmHg of LBNP will be applied for six minutes with thigh cuff inflation added the last three minutes. A minimum of 20 minutes will separate the two conditions.

The experimental protocol is illustrated in **Fig 1**. Prior to the start of the experiment, the subject will rest in a supine position for a minimum of 20 minutes to stabilize hemodynamic parameters. Manual arterial blood pressure will be measured using a brachial cuff, and heart rate will be measured by ECG to define LBNP stop criteria. After five minutes without LBNP, 60 mmHg LBNP will be applied for six minutes, either with or without the addition of inflated thigh cuffs for the last three minutes. After 20 minutes of supine rest, another five minutes of registration without LBNP followed by six minutes 60 mmHg LBNP will be repeated. During the second LBNP-exposure, the opposite thigh-cuff intervention is applied so that all subjects are exposed to LBNP with and without inflated thigh cuffs (crossover).

Fig 1. Illustration of the experimental protocol. *The subject will participate in two experimental conditions. In randomized order, the subject will participate in one condition including lower body negative pressure (LBNP) 60 mmHg for six minutes*

and another experimental condition with both LBNP 60 mmHg for six minutes and thigh cuff inflation for the last three minutes.

LBNP will be terminated after completing six minutes of 60 mmHg of LBNP or earlier if the subject exhibits signs or symptoms of hemodynamic decompensation, e.g. lightheadedness, nausea, or sweating. Objective stop criteria for early termination include a reduction in heart rate or MAP to less than 75% of baseline for more than three seconds. Additionally, LBNP will be terminated at the subject's request for any reason other than those aforementioned.

Subjects

Healthy volunteers will be included according to the inclusion and exclusion criteria in **Fig 2.** The subjects must refrain from caffeine (six hours), alcohol (24 hours) and strenuous exercise (on the test day) prior to the visit. They are allowed to eat a light meal until two hours beforehand. A thorough introduction of the experimental setup will be given before the subject provides written and oral consent to participate in the study. Each subject will receive a universal gift card of 500 NOK (approximately €43) to compensate for the inconvenience and discomfort of participating in the study.

Table 1. Inclusion and exclusion criteria for the study population.

**Inclusion criteria**
Age between 18 and 50 years
Normal physical performance (e.g., ability to walk and climb stairs without discomfort)
Able to provide consent

**Exclusion criteria**
History of syncope (except vasovagal syncope with known precipitating cause)
Conditions limiting physical performance or requiring regular medication (except for birth control or allergy medication)
History of arrhythmia
Pregnancy

Randomization

The subjects will be randomized in a ratio of 1:1 to begin the test session with one of the two experimental conditions: LBNP or LBNP and thigh cuff inflation. To ensure an approximately equal number of subjects beginning the test session with either experimental condition, we will use block randomization with blocks of four or eight using the "blockrand" package in R [18]. A person otherwise not involved in the conduct of the study will prepare sealed envelopes with sequential allocations.

Interventions

Lower body negative pressure

The subjects will be positioned in the supine position with deflated thigh cuffs and a saddle between the legs to prevent the subject from activating leg muscle and thereby the muscle vein pump. The chamber will be air sealed at the level of the iliac crest. The room temperature will be maintained between 22 and 24 °C. The model is illustrated in Error! Reference source not found..

Fig 2. ***Illustration showing the subject with thigh cuffs inside the LBNP chamber.*** *1) The lower body negative pressure (LBNP) chamber is 2) sealed just above the iliac crest and connected to 3) a vacuum pump controlled by 4) a pressure control unit. The applied negative pressure is displayed on 5) a pressure monitor. 6) Thigh cuffs are positioned proximal on both thighs, inflated by 7) Venopulse. 8) The pulse oximeter is positioned at the first toe on the left foot, ensuring complete occlusion to the lower limbs. Measuring 9) ascending aortic blood velocity by suprasternal pulsed Doppler ultrasound, 10) ECG, 11) mean arterial pressure (MAP) on 12) a data acquisition device, 13) sampled continuously on a laptop. 14) Cerebral tissue oxygen saturation (ScO2) will be measured by near-infrared spectroscopy sampled on 15) an Invos monitor. 16) Mean cerebral artery velocity is sampled by a 1-5 MHz M5S probe connected to 17) triplex transcranial ultrasound.*

Thigh cuffs

To approximate the hemodynamic effects of zone 3 REBOA, bilateral thigh cuffs will be positioned proximally on the thighs and inflated to a suprasystolic pressure during LBNP as described in **Fig 2.** The thigh cuffs will be inflated to 80 mmHg above systolic blood pressure, as given by the manual arterial blood pressure measured at rest before LBNP. To minimize discomfort of the cuffs, we will use contoured thigh cuffs (CC22, 24x122,5cm cuff size Hokanson, Bellevue, WA, United States) with careful attention given to find the best and most comfortable placement as proximal as possible on the thighs. The thigh cuffs will be inflated rapidly using a Rapid Cuff Inflation connected to Venopulse (STR Teknikk, Ålesund, Norway). Arterial occlusion will be confirmed by the disappearance of photoplethysmographic pulsation of a pulse oximeter placed on the first toe of the left foot.

Measurements and data processing

Ascending aortic blood velocity will be measured by suprasternal pulsed Doppler ultrasound using a 2 MHz probe (SD-50; Vingmed Ultrasound, Horten, Norway). Stroke volume will be calculated as the product of the aortic blood velocity-time integral and left ventricular

outflow tract (LVOT) area. The diameter of the LVOT will be measured with echocardiography in the parasternal long-axis (1-5 MHZ M5S probe, Vivid E95; GE Vingmed, Horten, Norway) [19], and the area will be calculated assuming a circular LVOT. The heart rate will be calculated from a three-lead ECG (Bio Amp/PowerLab, AD Instruments, Bella Vista, Australia). Cardiac output will be calculated as the product of heart rate and stroke volume. The arterial blood pressure waveform will be measured continuously and noninvasively using the volume clamp method on the third finger of the left hand (Human NIBP Nano System, Finapres Medical System B.V., Netherlands). End-tidal CO_2 ($ETCO_2$) will be measured using side stream capnography (Medlab CAP 10; Medlab GmbH, Stutensee, Germany). The photoplethysmography (PPG) waveform from the first toe on the left foot will be acquired from a pulse oximeter (Masimo Radical 7; Maximo Corp, CA, USA).

We will measure MCAV using triplex transcranial Doppler (TCD) ultrasound with a 1-5 MHz M5S probe (Vivid E95; GE Vingmed, Horten, Norway). The probe will be positioned over the right anterior temporal window and the middle cerebral artery (MCA) will be identified by its characteristic shape, depth (25-50mm), and blood flow direction [20]. The sample volume will be placed to obtain a clear Doppler signal with minimal insonation angle. The probe position, and ultrasound machine settings, including the sample volume and angle will not be changed during both experimental conditions within each subject. The same operator will perform all TCD measurements to ensure reliability and uniformity in data collection. Cerebral tissue oxygen saturation (ScO_2) will be obtained by near-infrared spectroscopy (Invos 5100C cerebral/somatic oximeter; Somanetics, Troy, MI, USA). Pain will be registered using a verbal numeric rating scale ranging from 0 (no pain) to 10 (worst imaginable pain).

Stroke volume, heart rate, cardiac output, MAP, $ETCO_2$ and PPG waveform will be sampled at 1000 Hz in Lab Chart 8.1.28 (ADInstruments, Bella Vista, Australia). MCAV

recordings will be exported as avi-files to Cardiovascular Suite 4.0 (Quipu, Pisa, Italy) for analysis of the Doppler waveform to calculate the time averaged maximum velocity and exported as .txt-files. To synchronize the timing of the TCD recordings with the other recorded signals, the recordings will be mirrored directly into Lab Chart 8.1.28 via Digital Visual Interface (DVI) using a video grabber. ScO_2 data will be exported as .txt-files. Analyses will be performed in R [21] / RStudio [22].

Outcomes

The primary outcome is the effect of thigh cuff inflation on stroke volume during LBNP. The secondary outcomes are the effects of thigh cuff inflation on MAP and MCAV during LBNP.

Statistical methods

We have previously observed a mean (standard deviation, SD) reduction in stroke volume of 44 (11) % during 60 mmHg compared to zero mmHg of LBNP [23]. Assuming this change, 16 subjects will detect a 10% difference between the experimental conditions with $\alpha=0.05$ and $(1-\beta)=0.90$. To account for potential dropouts, we aim to enroll 20 subjects.

Due to the repeated nature of the data and clustering within subjects, the pre-defined primary and secondary hypotheses will be evaluated using mixed regression models. The details in the models (e.g. covariance structures and use of generalized additive mixed models) will depend on the data (residual distribution and linearity).

Organization and conduct

This trial is approved by the Regional Ethics Committee (REK South-East D, ref. nr: 522416) and the Data Protection Officer at Oslo University Hospital (ref.nr: 22/22307). Sensitive data will be stored de-identified on a secure data server at Oslo University Hospital.

Discussion

In this present study, we will combine two established experimental interventions: LBNP and thigh cuff inflation. We thereby aim to explore if this combination can be used as a model in awake humans to elucidate the systemic and cerebral hemodynamic effects which can be seen with zone 3 REBOA in hemorrhage.

The hemodynamic response to REBOA in the non-cardiac arrest setting has been mainly described in animal models. In a normovolemic canine model, ligation of the descending aorta led to an increase in systolic blood pressure and end systolic- and end diastolic volumes of the left ventricle. Simultaneously, flow in the superior vena cava increased, indicating an increased flow to this vascular bed [24]. In a porcine model of hemorrhagic shock, REBOA increased proximal pressure, carotid artery blood flow, and brain oxygenation [25]. The combined effects of TBI and REBOA were studied in a rodent model. The setup and results were quite complex, but REBOA increased MAP and cerebral blood flow in the group with sham-TBI [26]. Although one pig model of TBI and hemorrhage did not find increased TBI-progression [27], the question of possible adverse effects of supranormal precerebral arterial pressures has been raised. In a registry study, systolic blood pressure >160 mmHg was associated with worse outcomes in TBI-patients [28], and a case report described fatal intracerebral hemorrhage in a patient treated with REBOA for a pelvic hemorrhage who had a concomitant TBI [29]. However, the effects on MAP were less for a zone 3 REBOA compared to zone 1 in a pig model with hemorrhage [30]. Performing a study on the effects on systemic and cerebral hemodynamic effects of hemorrhage and increased afterload in awake humans, therefore, seems justified.

Methodological considerations

Thigh cuff inflation will only occlude the vascular beds distal to the femoral arteries, missing the pelvis compared to a zone 3 REBOA. One could imagine occluding additional vascular beds, e.g. by manual compression of the abdominal aorta against the columna. However, at this point, we do not wish to impose excessive hemodynamic interventions due to the lack of experience in combining these two interventions when each in isolation includes quite profound hemodynamic effects. To the best of our knowledge, only one previous study has combined LBNP with arterial occlusion at thigh level [31] However, in that study, lower limb tourniquets were applied before exposure to LBNP, potentially limiting the redistribution of blood to the lower limbs below the tourniquets. In contrast to REBOA, which occludes only the arteries, the veins are also occluded in our and the tourniquet model. Although we do not fully know the consequences of additional venous occlusion, venous congestion is already present in the lower extremities due to LBNP. Therefore, we do not believe that this will preclude the model.

The application of thigh cuff inflation may cause pain, a known trigger of the sympathetic nervous system, which can increase arterial blood pressure [23]. This may confound the hemodynamic effects of vascular occlusion. However, as both arterial occlusion and increased sympathetic activity tend to increase blood pressure, pain will not counteract the intended effect of vascular occlusion. To minimize pain and discomfort, we will use contoured thigh cuffs designed for a better fit and more even pressure distribution. Additionally, we will assess pain associated with the cuffs using a verbal numerical rating scale to evaluate the severity of discomfort and explore its impact. It is not feasible to blind neither the subjects nor the investigators for thigh cuffs. Occlusion lasting a period as short

as three minutes is within the safe inflation time of 1,5-2 hours, rarely associated with complications [16].

LBNP has been used for several decades and is believed to represent minimal risk in healthy humans [15]. There are, however, differences between traumatic and simulated hemorrhage, as traumatic hemorrhage may also include tissue damage leading to complex genomic, cellular, tissue and organ alterations [32]. In this present study, the subjects will be exposed to simulated hemorrhage for six minutes in each experimental condition, shorter than most clinical hemorrhagic episodes.

We will use suprasternal Doppler ultrasound to obtain stroke volume [19]. It has been shown that the systolic velocity profile in the LVOT stays rectangular with little change in velocity from the aortic orifice for 3-4 cm above the aortic valve, even if the aortic diameter changes slightly [19]. Thus, the sampling volume may be positioned in the ascending aorta to minimize noise and improve the quality of the Doppler signal without reducing the accuracy [19]. The 2 MHz probe will be positioned in the suprasternal notch, giving a fixed angle on insonation during LBNP [33]. TCD provides blood velocity of the MCA, which reflects cerebral blood flow, assuming a constant caliber of the vessel [20,34].

For safety reasons, LBNP and thigh occlusion will not be released simultaneously. Thigh occlusion will be relieved 30 seconds after LBNP has been released to prevent excessive hypotension that could arise from relieving thigh occlusion before the effects of LBNP have subsided.

In conclusion, the present study aims to combine LBNP and thigh cuff inflation to investigate the systemic and cerebral hemodynamic effects of thigh cuff inflation during LBNP in healthy volunteers.

Trial status

Data collection started in April 2024 and is expected to be completed in February 2025. The results will be submitted for publishing in a peer-reviewed international journal and presented at scientific conferences.

Author Contributions

Conceptualization: Sara Stadskleiv Torbjørnsen, Sole Lindvåg Lie, Jonny Hisdal, Marius Rehn, Lars Øivind Høiseth

Methodology: Sara Stadskleiv Torbjørnsen, Sole Lindvåg Lie, Jonny Hisdal, Marius Rehn, Lars Øivind Høiseth

Project administration: Sara Stadskleiv Torbjørnsen, Sole Lindvåg Lie, Lars Øivind Høiseth

Supervision: Sole Lindvåg Lie, Jonny Hisdal, Marius Rehn, Lars Øivind Høiseth

Writing – original draft: Sara Stadskleiv Torbjørnsen

Writing – review & editing: Sole Lindvåg Lie, Jonny Hisdal, Marius Rehn, Lars Øivind Høiseth

SLL, JH and LØH conceived and designed the research. SST and SLL will conduct data collection and SST, SLL and LØH will do the data analyses. All authors will contribute to the interpretation of the data. SST drafted this manuscript. SLL, JH, MR and LØH have provided feedback. All authors have read and approved the final version of this protocol manuscript.

References

1. Kauvar DS, Lefering R, Wade CE. Impact of Hemorrhage on Trauma Outcome: An Overview of Epidemiology, Clinical Presentations, and Therapeutic Considerations. J. Trauma Acute Care Surg. 2006;60:S3.

2. Gomez D, Berube M, Xiong W, Ahmed N, Haas B, Schuurman N, et al. Identifying Targets for Potential Interventions to Reduce Rural Trauma Deaths: A Population-Based Analysis. J. Trauma Inj. Infect. Crit. Care 2010;69:633–9.

3. Eastridge BJ, Holcomb JB, Shackelford S. Outcomes of traumatic hemorrhagic shock and the epidemiology of preventable death from injury. Transfusion (Paris) 2019;59:1423–8.

4. Kotwal RS, Montgomery HR, Kotwal BM, Champion HR, Butler FK, Mabry RL, et al. Eliminating Preventable Death on the Battlefield. Arch. Surg. 2011;146:1350–8.

5. Nunez RM, Naranjo MP, Foianini E, Ferrada P, Rincon E, Garcia-Perdomo HA, et al. A meta-analysis of resuscitative endovascular balloon occlusion of the aorta (REBOA) or open aortic cross-clamping by resuscitative thoracotomy in non-compressible torso hemorrhage patients. World J. Emerg. Surg. [Internet] 2017 [cited 2024 Dec 9];12. Available from: https://go-gale-com.ezproxy.uio.no/ps/i.do?p=AONE&sw=w&issn=17497922&v=2.1&it=r&id=GALE%7CA511353344&sid=googleScholar&linkaccess=abs

6. Thrailkill MA, Gladin KH, Thorpe CR, Roberts TR, Choi JH, Chung KK, et al. Resuscitative Endovascular Balloon Occlusion of the Aorta (REBOA): update and insights into current practices and future directions for research and implementation. Scand. J. Trauma Resusc. Emerg. Med. 2021;29:8.

7. Sadek S, Lockey DJ, Lendrum RA, Perkins Z, Price J, Davies GE. Resuscitative endovascular balloon occlusion of the aorta (REBOA) in the pre-hospital setting: An additional resuscitation option for uncontrolled catastrophic haemorrhage. Resuscitation 2016;107:135–8.

8. Hughes CW. Use of an intra-aortic balloon catheter tamponade for controlling intra-abdominal hemorrhage in man. Surgery 1954;36:65–58.

9. Moore LJ, Brenner M, Kozar RA, Pasley J, Wade CE, Baraniuk MS, et al. Implementation of resuscitative endovascular balloon occlusion of the aorta as an alternative to resuscitative thoracotomy for noncompressible truncal hemorrhage. J. Trauma Acute Care Surg. 2015;79:523–32.

10. Stonko DP, Edwards J, Abdou H, Elansary NN, Lang E, Savidge SG, et al. The Underlying Cardiovascular Mechanisms of Resuscitation and Injury of REBOA and Partial REBOA. Front. Physiol. [Internet] 2022 [cited 2024 Dec 10];13. Available from: https://www.frontiersin.org/journals/physiology/articles/10.3389/fphys.2022.871073/full

11. Russo RM, White JM, Baer DG. Partial Resuscitative Endovascular Balloon Occlusion of the Aorta: A Systematic Review of the Preclinical and Clinical Literature. J. Surg. Res. 2021;262:101–14.

12. Jha RM, Shutter L. Neurologic complications of polytrauma [Internet]. In: Handbook of Clinical Neurology. Elsevier; 2017 [cited 2024 Dec 10]. page 633–55.Available from: https://linkinghub.elsevier.com/retrieve/pii/B978044463599000034X

13. Gross T, Schüepp M, Attenberger C, Pargger H, Amsler F. Outcome in polytraumatized patients with and without brain injury. Acta Anaesthesiol. Scand. 2012;56:1163–74.

14. Belenkiy SM, Batchinsky AI, Rasmussen TE, Cancio LC. Resuscitative endovascular balloon occlusion of the aorta for hemorrhage control: Past, present, and future. J. Trauma Acute Care Surg. 2015;79:S236–42.

15. Goswami N, Blaber AP, Hinghofer-Szalkay H, Convertino VA. Lower Body Negative Pressure: Physiological Effects, Applications, and Implementation. Physiol. Rev. 2019;99:807–51.

16. Deloughry JL, Griffiths R. Arterial tourniquets. Contin. Educ. Anaesth. Crit. Care Pain 2009;9:56–60.

17. Marcinkevics Z, Rubins U, Zelca UU, Obuhovskis D, Grabovskis A. Impact of Bilateral Thigh Occlusion on the Remote Photoplethysmography Signal Recorded from the Palm [Internet]. In: 2024 IEEE 14th International Conference Nanomaterials: Applications & Properties (NAP). Riga, Latvia: IEEE; 2024 [cited 2024 Dec 12]. page 1–5.Available from: https://ieeexplore.ieee.org/document/10739727/

18. Snow G. Blockrand: Randomization for Block Random Clinical Trials [Internet]. 2020;Available from: https://CRAN.R-project.org/package=blockrand

19. Eriksen M, Walløe L. Improved method for cardiac output determination in man using ultrasound Doppler technique. Med. Biol. Eng. Comput. 1990;28:555–60.

20. Willie CK, Colino FL, Bailey DM, Tzeng YC, Binsted G, Jones LW, et al. Utility of transcranial Doppler ultrasound for the integrative assessment of cerebrovascular function. J. Neurosci. Methods 2011;196:221–37.

21. R Core Team. R: A Language and Environment for Statistical Computing [Internet]. 2021;Available from: https://www.R-project.org/

22. RStudio Team RStudio. Integrated Development Environment for R. 2020;

23. Høiseth LØ, Hisdal J, Hoff IE, Hagen OA, Landsverk SA, Kirkebøen KA. Tissue oxygen saturation and finger perfusion index in central hypovolemia: influence of pain. Crit. Care Med. 2015;43:747–56.

24. Stokland O, Miller MM, Ilebekk A, Kiil F. Mechanism of hemodynamic responses to occlusion of the descending thoracic aorta. Am. J. Physiol.-Heart Circ. Physiol. 1980;238:H423–9.

25. White JM, Cannon JW, Stannard A, Markov NP, Spencer JR, Rasmussen TE. Endovascular balloon occlusion of the aorta is superior to resuscitative thoracotomy with aortic clamping in a porcine model of hemorrhagic shock. Surgery 2011;150:400–9.

26. Bailey ZS, Cardiff K, Yang X, Gilsdorf J, Shear D, Rasmussen TE, et al. The Effects of Balloon Occlusion of the Aorta on Cerebral Blood Flow, Intracranial Pressure, and Brain

Tissue Oxygen Tension in a Rodent Model of Penetrating Ballistic-Like Brain Injury. Front. Neurol. 2019;10:1309.

27. Johnson MA, Williams TK, Ferencz SAE, Davidson AJ, Russo RM, O’Brien WT, et al. The Effect of REBOA, Partial Aortic Occlusion and Aggressive Blood Transfusion on Traumatic Brain Injury in a Swine Polytrauma Model. J. Trauma Acute Care Surg. 2017;83:61–70.

28. Sellmann T, Miersch D, Kienbaum P, Flohé S, Schneppendahl J, Lefering R, et al. The impact of arterial hypertension on polytrauma and traumatic brain injury. Dtsch. Arzteblatt Int. 2012;109:849–56.

29. Uchino H, Tamura N, Echigoya R, Ikegami T, Fukuoka T. “REBOA” – Is it Really Safe? A Case with Massive Intracranial Hemorrhage Possibly due to Endovascular Balloon Occlusion of the Aorta (REBOA). Am. J. Case Rep. 2016;17:810–3.

30. Tibbits EM, Hoareau GL, Simon MA, Davidson AJ, DeSoucy ES, Faulconer ER, et al. Location is everything: The hemodynamic effects of REBOA in Zone 1 versus Zone 3 of the aorta. J. Trauma Acute Care Surg. 2018;85:101–7.

31. Hutchings SD, Watchorn J, McDonald R, Jeffreys S, Bates M, Watts S, et al. Quantification of stroke volume in a simulated healthy volunteer model of traumatic haemorrhage; a comparison of two non-invasive monitoring devices using error grid analysis alongside traditional measures of agreement. PloS One 2021;16:e0261546.

32. Latif RK, Clifford SP, Baker JA, Lenhardt R, Haq MZ, Huang J, et al. Traumatic hemorrhage and chain of survival. Scand. J. Trauma Resusc. Emerg. Med. 2023;31:25.

33. Taylor JA, Halliwill JR, Brown TE, Hayano J, Eckberg DL. “Non-hypotensive” hypovolaemia reduces ascending aortic dimensions in humans. J. Physiol. 1995;483 (Pt 1):289–98.

34. Ainslie PN, Hoiland RL. Transcranial Doppler ultrasound: Valid, invalid, or both? J. Appl. Physiol. 2014;117:1081–3.

Figure 1

Click here to access/download;Figure;Fig1.tiff

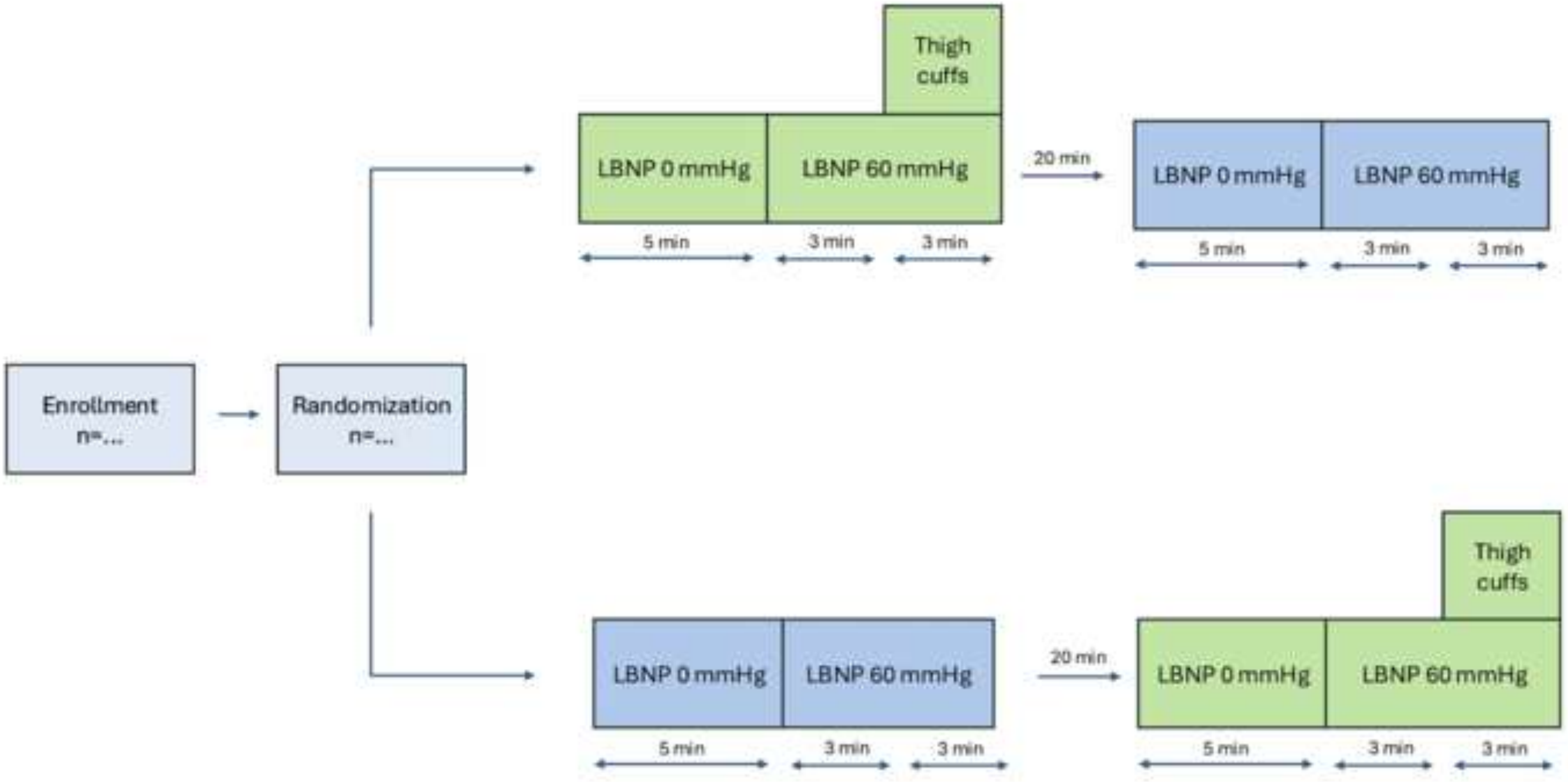

Figure 2

Click here to access/download;Figure;Fig2.tiff

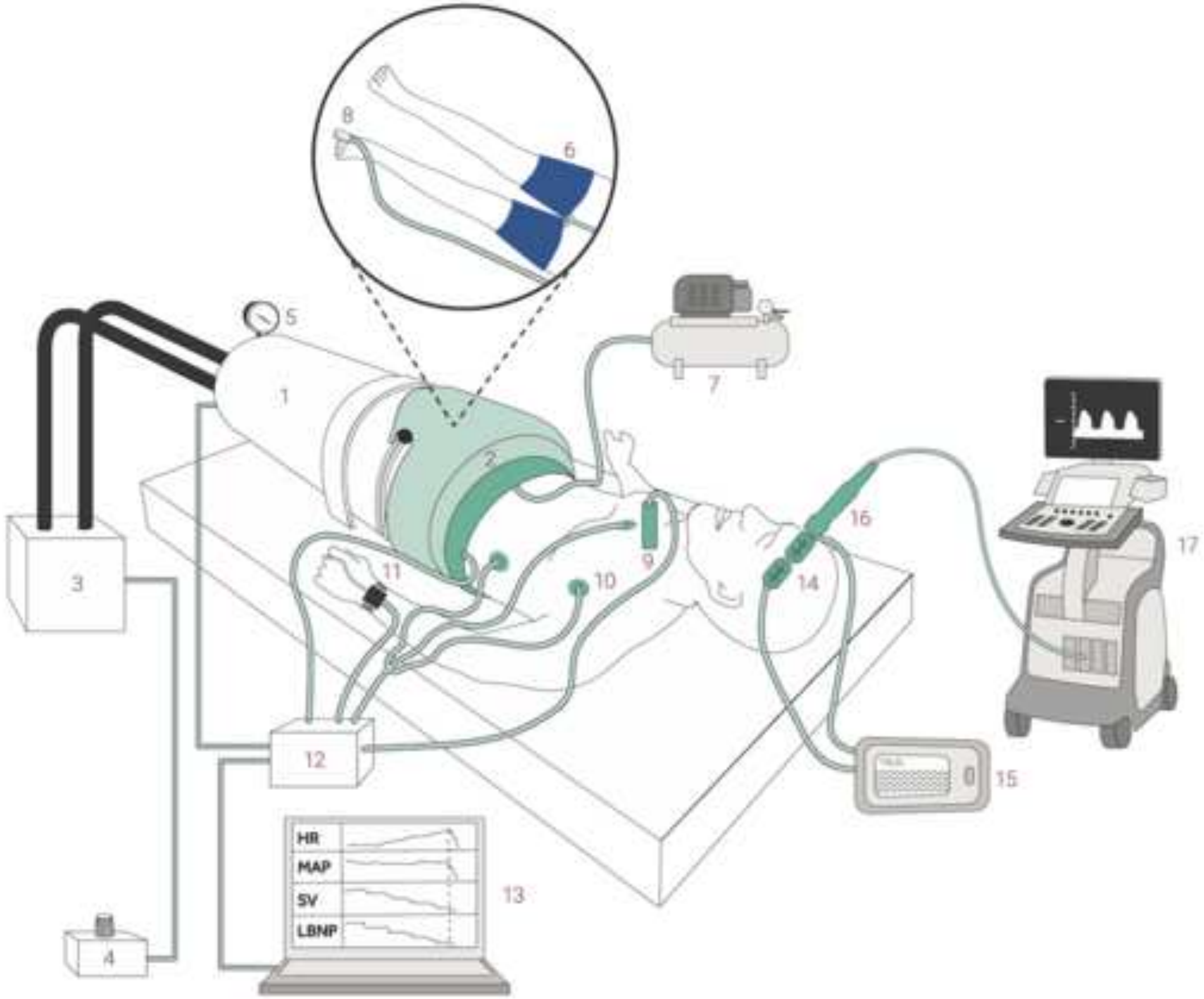

Supplement: Attachment — Submitted filename: PONE-D-24-58304 coloured.pdf [file pone.0327404.s001.pdf]
